# 5,5′-Bis[(1*H*-imidazol-1-yl)meth­yl]-2,2′-bipyridine methanol disolvate

**DOI:** 10.1107/S1600536811003643

**Published:** 2011-02-02

**Authors:** Suk-Hee Moon, Tae Ho Kim, Ki-Min Park

**Affiliations:** a Department of Food & Nutrition, Kyungnam College of Information and Technology, Busan 616-701, Republic of Korea; b Department of Chemistry and Research Institute of Natural Sciences, Gyeongsang National University, Jinju 660-701, Republic of Korea

## Abstract

The title compound, C_18_H_16_N_6_·2CH_3_OH, was prepared by the reaction of 5,5′-bis­(bromo­meth­yl)-2,2′-bipyridine with imidazole. The main mol­ecule lies on an inversion center located at the mid-point of the C—C bond joining the two pyridine rings. The asymmetric unit therefore contains one half-mol­ecule and one methanol solvent mol­ecule. The dihedral angle between the pyridine and imidazole rings is 72.32 (5)°. In the crystal, weak inter­molecular O—H⋯N, C—H⋯N and C—H⋯O hydrogen bonds contribute to the stabilization of the packing.

## Related literature

For related syntheses, see: Sambrook *et al.* (2006 ▶); Zang *et al.* (2010 ▶). For a related structure, see: Zang *et al.* (2010 ▶). For reference bond lengths, see: Allen *et al.* (1987 ▶).
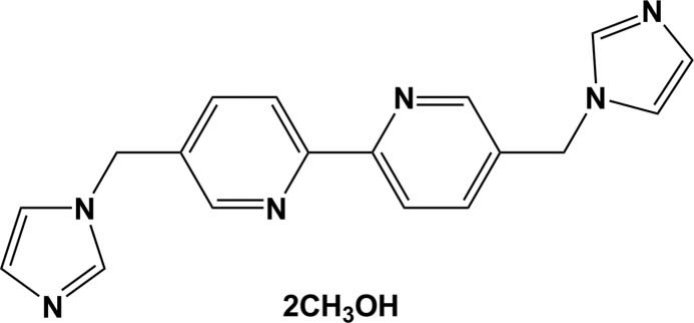


## Experimental

### 

#### Crystal data

C_18_H_16_N_6_·2CH_4_O
*M* *_r_* = 380.45
Monoclinic, 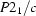

*a* = 4.5653 (4) Å
*b* = 14.7886 (12) Å
*c* = 14.5378 (11) Å
β = 93.805 (2)°
*V* = 979.35 (14) Å^3^
*Z* = 2
Mo *K*α radiation
μ = 0.09 mm^−1^
*T* = 173 K
0.35 × 0.30 × 0.10 mm

#### Data collection

Bruker APEXII CCD diffractometer
Absorption correction: multi-scan (*SADABS*; Sheldrick, 1996 ▶) *T*_min_ = 0.970, *T*_max_ = 0.991
5948 measured reflections
2136 independent reflections
1619 reflections with *I* > 2σ(*I*)
*R*_int_ = 0.053

#### Refinement

*R*[*F* ^2^ > 2σ(*F* ^2^)] = 0.041
*wR*(*F* ^2^) = 0.144
*S* = 1.09
2136 reflections
128 parameters
H-atom parameters constrained
Δρ_max_ = 0.21 e Å^−3^
Δρ_min_ = −0.24 e Å^−3^

### 

Data collection: *APEX2* (Bruker, 2006 ▶); cell refinement: *SAINT* (Bruker, 2006 ▶); data reduction: *SAINT*; program(s) used to solve structure: *SHELXTL* (Sheldrick, 2008 ▶); program(s) used to refine structure: *SHELXTL*; molecular graphics: *SHELXTL* and *DIAMOND* (Brandenburg, 1998 ▶); software used to prepare material for publication: *SHELXTL*.

## Supplementary Material

Crystal structure: contains datablocks I, global. DOI: 10.1107/S1600536811003643/sj5094sup1.cif

Structure factors: contains datablocks I. DOI: 10.1107/S1600536811003643/sj5094Isup2.hkl

Additional supplementary materials:  crystallographic information; 3D view; checkCIF report

## Figures and Tables

**Table 1:** Hydrogen-bond geometry (Å, °)

*D*—H⋯*A*	*D*—H	H⋯*A*	*D*⋯*A*	*D*—H⋯*A*
O1—H1*A*⋯N3^i^	0.84	1.93	2.7662 (19)	171
C3—H3⋯O1	0.95	2.44	3.360 (2)	162
C8—H8⋯N1^ii^	0.95	2.57	3.422 (2)	149
C9—H9⋯O1^iii^	0.95	2.54	3.470 (2)	168

Symmetry codes: (i) 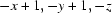; (ii) 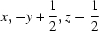; (iii) 
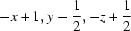
.

**Fig. 1.**
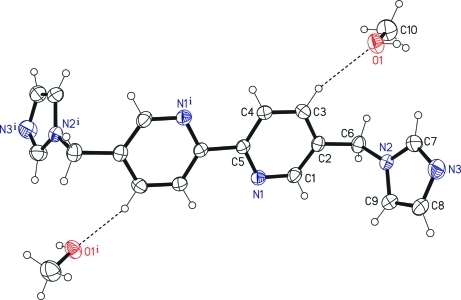
The molecular structure of the title compound, showing displacement ellipsoids drawn at the 50% probability level. Hydrogen bonds are shown as dashed lines. (Symmetry code: i) -x + 2, -y + 1, -z + 1)

**Fig. 2.**
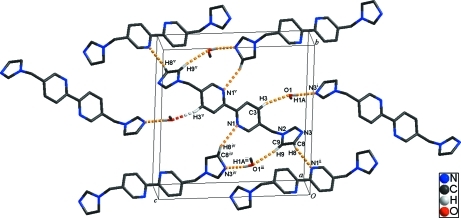
Crystal packing of the title compound with intermolecular O–H···N, C–H···N and C–H···O hydrogen bonds shown as dashed lines. H atoms not involved in intermolecular interactions have been omitted for clarity (Symmetry codes: i) -x + 1, -y + 1, -z; ii) x, -y + 1/2, z - 1/2; iii) -x + 1, y - 1/2, -z + 1/2; iv) x, -y + 1/2, z + 1/2; v) -x + 2, -y + 1, -z + 1).

## supplementary crystallographic information

### Comment

The title compound was prepared to use as a multi-dentate ligand in the formation of metallosupramolecules in line with similar previously reported compounds (Sambrook *et al.*, 2006; Zang *et al.*, 2010).

In the title compound (Scheme 1, Fig. 1), two pyridine rings are coplanar because the title compound lies on a crystallographic inversion center. The dihedral angle between the pyridine and imidazole rings is 72.32 (5)°. All the bond lengths are within normal values (Allen *et al.*, 1987).

In the crystal structure, as shown in Fig. 2, weak intermolecular O–H···N, C–H···N and C–H···O hydrogen bonds are observed (Table 1). These intermolecular interactions may be contribute to the stabilization of the packing.

### Experimental

A mixture of imidazole (0.120 g, 1.76 mmol) and potassium hydroxide (0.440 g, 7.84 mmol) in DMSO (10 ml) was stirred for 1 h. A DMSO solution (20 ml) of 5,5'-bis(bromomethyl)-2,2'-bipyridine (0.30 g, 0.88 mmol) was slowly added and the solution stirred for 6 h at room temperature. After water (100 ml) was added, the reaction mixture was extracted with chloroform (3×100 ml), washed with water and then dried over anhydrous MgSO_4_. The solvent was removed to give the title compound in 63% yield. X-ray quality single crystals were obtained by slow evaporation of a solution in MeOH.

### Refinement

All H-atoms were positioned geometrically and refined using a riding model with d(C—H) = 0.95 Å, *U*_iso_ = 1.2*U*_eq_(C) for aromatic, d(C—H) = 0.84 Å, *U*_iso_ = 1.5*U*_eq_(C) for hydroxyl, d(C—H) = 0.98 Å, *U*_iso_ = 1.5*U*_eq_(C) for methyl protons.

### Crystal data

**Table:**

C_18_H_16_N_6_·2CH_4_O	*F*(000) = 404
*M**_r_* = 380.45	*D*_x_ = 1.290 Mg m^−^^3^
Monoclinic, *P*2_1_/*c*	Mo *K*α radiation, λ = 0.71073 Å
Hall symbol: -P 2ybc	Cell parameters from 2441 reflections
*a* = 4.5653 (4) Å	θ = 2.8–28.2°
*b* = 14.7886 (12) Å	µ = 0.09 mm^−^^1^
*c* = 14.5378 (11) Å	*T* = 173 K
β = 93.805 (2)°	Plate, colorless
*V* = 979.35 (14) Å^3^	0.35 × 0.30 × 0.10 mm
*Z* = 2	

### Data collection

**Table:**

Bruker APEXII CCD diffractometer	2136 independent reflections
Radiation source: fine-focus sealed tube	1619 reflections with *I* > 2σ(*I*)
graphite	*R*_int_ = 0.053
φ and ω scans	θ_max_ = 27.0°, θ_min_ = 2.0°
Absorption correction: multi-scan (*SADABS*; Sheldrick, 1996)	*h* = −4→5
*T*_min_ = 0.970, *T*_max_ = 0.991	*k* = −18→18
5948 measured reflections	*l* = −16→18

### Refinement

**Table:**

Refinement on *F*^2^	Primary atom site location: structure-invariant direct methods
Least-squares matrix: full	Secondary atom site location: difference Fourier map
*R*[*F*^2^ > 2σ(*F*^2^)] = 0.041	Hydrogen site location: inferred from neighbouring sites
*wR*(*F*^2^) = 0.144	H-atom parameters constrained
*S* = 1.09	*w* = 1/[σ^2^(*F*_o_^2^) + (0.0772*P*)^2^ + 0.1442*P*] where *P* = (*F*_o_^2^ + 2*F*_c_^2^)/3
2136 reflections	(Δ/σ)_max_ < 0.001
128 parameters	Δρ_max_ = 0.21 e Å^−^^3^
0 restraints	Δρ_min_ = −0.24 e Å^−^^3^

### Special details

**Table:**

Geometry. All e.s.d.'s (except the e.s.d. in the dihedral angle between two l.s. planes) are estimated using the full covariance matrix. The cell e.s.d.'s are taken into account individually in the estimation of e.s.d.'s in distances, angles and torsion angles; correlations between e.s.d.'s in cell parameters are only used when they are defined by crystal symmetry. An approximate (isotropic) treatment of cell e.s.d.'s is used for estimating e.s.d.'s involving l.s. planes.
Refinement. Refinement of *F*^2^ against ALL reflections. The weighted *R*-factor *wR* and goodness of fit *S* are based on *F*^2^, conventional *R*-factors *R* are based on *F*, with *F* set to zero for negative *F*^2^. The threshold expression of *F*^2^ > σ(*F*^2^) is used only for calculating *R*-factors(gt) *etc*. and is not relevant to the choice of reflections for refinement. *R*-factors based on *F*^2^ are statistically about twice as large as those based on *F*, and *R*- factors based on ALL data will be even larger.

### Fractional atomic coordinates and isotropic or equivalent isotropic displacement parameters (Å^2^)

**Table:**

	*x*	*y*	*z*	*U*_iso_*/*U*_eq_	
N1	0.8022 (3)	0.40136 (8)	0.45831 (9)	0.0287 (3)	
N2	0.4002 (3)	0.36956 (9)	0.17390 (9)	0.0281 (3)	
N3	0.5877 (4)	0.36182 (11)	0.03818 (10)	0.0441 (4)	
C1	0.5974 (4)	0.37871 (10)	0.39254 (11)	0.0301 (4)	
H1	0.5329	0.3176	0.3901	0.036*	
C2	0.4722 (3)	0.43832 (10)	0.32748 (10)	0.0254 (4)	
C3	0.5628 (4)	0.52770 (11)	0.33297 (11)	0.0302 (4)	
H3	0.4831	0.5710	0.2900	0.036*	
C4	0.7704 (4)	0.55308 (10)	0.40161 (11)	0.0290 (4)	
H4	0.8328	0.6143	0.4070	0.035*	
C5	0.8868 (3)	0.48825 (9)	0.46263 (10)	0.0230 (3)	
C6	0.2535 (4)	0.40622 (11)	0.25242 (11)	0.0309 (4)	
H6B	0.1273	0.3589	0.2774	0.037*	
H6A	0.1260	0.4573	0.2313	0.037*	
C7	0.4342 (4)	0.41095 (12)	0.09281 (12)	0.0370 (4)	
H7	0.3563	0.4689	0.0770	0.044*	
C8	0.6558 (4)	0.28448 (12)	0.08778 (13)	0.0408 (5)	
H8	0.7665	0.2353	0.0663	0.049*	
C9	0.5437 (4)	0.28818 (11)	0.17102 (12)	0.0357 (4)	
H9	0.5604	0.2436	0.2181	0.043*	
O1	0.2950 (3)	0.64181 (9)	0.14603 (8)	0.0371 (3)	
H1A	0.3447	0.6360	0.0917	0.056*	
C10	−0.0113 (4)	0.65194 (15)	0.14519 (15)	0.0484 (5)	
H10B	−0.0687	0.6604	0.2084	0.073*	
H10A	−0.1069	0.5977	0.1186	0.073*	
H10C	−0.0717	0.7048	0.1080	0.073*	

### Atomic displacement parameters (Å^2^)

**Table:**

	*U*^11^	*U*^22^	*U*^33^	*U*^12^	*U*^13^	*U*^23^
N1	0.0345 (8)	0.0239 (7)	0.0274 (7)	−0.0042 (5)	0.0002 (6)	0.0016 (5)
N2	0.0318 (8)	0.0278 (7)	0.0244 (7)	0.0002 (5)	0.0000 (6)	−0.0046 (5)
N3	0.0533 (10)	0.0507 (9)	0.0289 (8)	0.0131 (8)	0.0063 (7)	−0.0033 (7)
C1	0.0367 (9)	0.0246 (8)	0.0290 (8)	−0.0063 (6)	0.0016 (7)	−0.0023 (6)
C2	0.0250 (8)	0.0304 (8)	0.0215 (7)	0.0000 (6)	0.0065 (6)	−0.0043 (6)
C3	0.0337 (9)	0.0288 (8)	0.0279 (8)	0.0038 (7)	−0.0008 (7)	0.0034 (6)
C4	0.0351 (9)	0.0219 (8)	0.0296 (8)	−0.0011 (6)	−0.0009 (7)	0.0008 (6)
C5	0.0256 (8)	0.0240 (7)	0.0201 (7)	0.0001 (6)	0.0065 (6)	−0.0015 (5)
C6	0.0267 (9)	0.0379 (9)	0.0283 (9)	−0.0004 (6)	0.0034 (7)	−0.0065 (7)
C7	0.0471 (11)	0.0361 (9)	0.0280 (9)	0.0088 (8)	0.0030 (8)	0.0007 (7)
C8	0.0483 (11)	0.0390 (10)	0.0345 (10)	0.0132 (8)	−0.0015 (8)	−0.0112 (8)
C9	0.0456 (11)	0.0273 (8)	0.0337 (9)	0.0037 (7)	−0.0014 (8)	−0.0043 (7)
O1	0.0342 (7)	0.0493 (7)	0.0277 (6)	0.0074 (5)	0.0024 (5)	0.0017 (5)
C10	0.0350 (10)	0.0590 (13)	0.0515 (12)	0.0067 (9)	0.0053 (9)	−0.0012 (9)

### Geometric parameters (Å, °)

**Table:**

N1—C1	1.335 (2)	C4—H4	0.9500
N1—C5	1.3420 (19)	C5—C5^i^	1.490 (3)
N2—C7	1.346 (2)	C6—H6B	0.9900
N2—C9	1.372 (2)	C6—H6A	0.9900
N2—C6	1.4653 (19)	C7—H7	0.9500
N3—C7	1.313 (2)	C8—C9	1.346 (3)
N3—C8	1.377 (2)	C8—H8	0.9500
C1—C2	1.388 (2)	C9—H9	0.9500
C1—H1	0.9500	O1—C10	1.406 (2)
C2—C3	1.386 (2)	O1—H1A	0.8400
C2—C6	1.506 (2)	C10—H10B	0.9800
C3—C4	1.382 (2)	C10—H10A	0.9800
C3—H3	0.9500	C10—H10C	0.9800
C4—C5	1.388 (2)		
			
C1—N1—C5	117.36 (14)	N2—C6—H6B	109.3
C7—N2—C9	106.80 (14)	C2—C6—H6B	109.3
C7—N2—C6	126.80 (14)	N2—C6—H6A	109.3
C9—N2—C6	126.30 (14)	C2—C6—H6A	109.3
C7—N3—C8	104.71 (15)	H6B—C6—H6A	108.0
N1—C1—C2	124.46 (14)	N3—C7—N2	112.04 (16)
N1—C1—H1	117.8	N3—C7—H7	124.0
C2—C1—H1	117.8	N2—C7—H7	124.0
C3—C2—C1	117.31 (15)	C9—C8—N3	110.55 (15)
C3—C2—C6	121.56 (15)	C9—C8—H8	124.7
C1—C2—C6	121.11 (14)	N3—C8—H8	124.7
C4—C3—C2	119.24 (15)	C8—C9—N2	105.89 (15)
C4—C3—H3	120.4	C8—C9—H9	127.1
C2—C3—H3	120.4	N2—C9—H9	127.1
C3—C4—C5	119.28 (14)	C10—O1—H1A	109.5
C3—C4—H4	120.4	O1—C10—H10B	109.5
C5—C4—H4	120.4	O1—C10—H10A	109.5
N1—C5—C4	122.32 (14)	H10B—C10—H10A	109.5
N1—C5—C5^i^	116.18 (16)	O1—C10—H10C	109.5
C4—C5—C5^i^	121.50 (16)	H10B—C10—H10C	109.5
N2—C6—C2	111.44 (13)	H10A—C10—H10C	109.5
			
C5—N1—C1—C2	1.6 (2)	C9—N2—C6—C2	73.5 (2)
N1—C1—C2—C3	−1.5 (2)	C3—C2—C6—N2	94.07 (17)
N1—C1—C2—C6	176.79 (15)	C1—C2—C6—N2	−84.11 (18)
C1—C2—C3—C4	0.1 (2)	C8—N3—C7—N2	−0.1 (2)
C6—C2—C3—C4	−178.13 (14)	C9—N2—C7—N3	0.3 (2)
C2—C3—C4—C5	1.0 (2)	C6—N2—C7—N3	177.02 (15)
C1—N1—C5—C4	−0.3 (2)	C7—N3—C8—C9	−0.1 (2)
C1—N1—C5—C5^i^	179.33 (15)	N3—C8—C9—N2	0.3 (2)
C3—C4—C5—N1	−0.9 (2)	C7—N2—C9—C8	−0.3 (2)
C3—C4—C5—C5^i^	179.44 (16)	C6—N2—C9—C8	−177.10 (16)
C7—N2—C6—C2	−102.61 (19)		

Symmetry codes: (i) −*x*+2, −*y*+1, −*z*+1.

### Hydrogen-bond geometry (Å, °)

**Table:**

*D*—H···*A*	*D*—H	H···*A*	*D*···*A*	*D*—H···*A*
O1—H1A···N3^ii^	0.84	1.93	2.7662 (19)	171
C3—H3···O1	0.95	2.44	3.360 (2)	162
C8—H8···N1^iii^	0.95	2.57	3.422 (2)	149
C9—H9···O1^iv^	0.95	2.54	3.470 (2)	168

Symmetry codes: (ii) −*x*+1, −*y*+1, −*z*; (iii) *x*, −*y*+1/2, *z*−1/2; (iv) −*x*+1, *y*−1/2, −*z*+1/2.
